# Histone deacetylase (HDAC) inhibition improves myocardial function and prevents cardiac remodeling in diabetic mice

**DOI:** 10.1186/s12933-015-0262-8

**Published:** 2015-08-07

**Authors:** Youfang Chen, Jianfeng Du, Yu Tina Zhao, Ling Zhang, Guorong Lv, Shougang Zhuang, Gangjian Qin, Ting C Zhao

**Affiliations:** Department of Surgery, Boston University Medical School, Roger Williams Medical Center, Boston University, 50 Maude Street, Providence, RI 02908 USA; Department of Medicine, Rhode Island Hospital, Brown University, Providence, RI USA; Department of Ultrasound, Second Affiliated Hospital of Fujian Medical University, Quanzhou, Fujian China; Feinberg Cardiovascular Research Institute, Northwestern University Feinberg School of Medicine, Chicago, USA

**Keywords:** HDAC, Diabetes, Myocardium, Heart failure, Apoptosis, GLUT

## Abstract

**Background:**

Recent evidence indicates that inhibition of histone deacetylase (HDAC) protects the heart against myocardial injury and stimulates endogenous angiomyogenesis. However, it remains unknown whether HDAC inhibition produces the protective effect in the diabetic heart. We sought to determine whether HDAC inhibition preserves cardiac performance and suppresses cardiac remodeling in diabetic cardiomyopathy.

**Methods:**

Adult ICR mice received an intraperitoneal injection of either streptozotocin (STZ, 200 mg/kg) to establish the diabetic model or vehicle to serve as control. Once hyperglycemia was confirmed, diabetic mice received sodium butyrate (1%), a specific HDAC inhibitor, in drinking water on a daily basis to inhibit HDAC activity. Mice were randomly divided into following groups, which includes *Control*, *Control* + *Sodium butyrate* (*NaBu*)*, STZ and STZ* + *Sodium butyrate* (*NaBu*), respectively. Myocardial function was serially assessed at 7, 14, 21 weeks following treatments.

**Results:**

Echocardiography demonstrated that cardiac function was depressed in diabetic mice, but HDAC inhibition resulted in a significant functional improvement in STZ-injected mice. Likewise, HDAC inhibition attenuates cardiac hypertrophy, as evidenced by a reduced heart/tibia ratio and areas of cardiomyocytes, which is associated with reduced interstitial fibrosis and decreases in active caspase-3 and apoptotic stainings, but also increased angiogenesis in diabetic myocardium. Notably, glucose transporters (GLUT) 1 and 4 were up-regulated following HDAC inhibition, which was accompanied with increases of GLUT1 acetylation and p38 phosphorylation. Furthermore, myocardial superoxide dismutase, an important antioxidant, was elevated following HDAC inhibition in the diabetic mice.

**Conclusion:**

HDAC inhibition plays a critical role in improving cardiac function and suppressing myocardial remodeling in diabetic heart.

**Electronic supplementary material:**

The online version of this article (doi:10.1186/s12933-015-0262-8) contains supplementary material, which is available to authorized users.

## Background

Cardiovascular complications are the leading cause of diabetes-related morbidity and mortality worldwide [1]. Diabetes mellitus threatens to become a global health crisis; treatment of diabetes and its complications constitutes a major health care expenditure. A significant proportion of diabetic patients are known to develop diabetic cardiomyopathy, with a high incidence of congestive heart failure [2–4]. For example, in patients with diabetes mellitus, both the prevalence and severity of manifestations of coronary artery disease are increased compared with non-diabetic subjects. Experimental evidence of diabetic cardiomyopathy has been accumulated in disease models with both type 1 and type 2 diabetes [5–8]. Evidence from STZ-induced hyperglycemia displayed decreased cardiac function, a reduction in cardiac mass over time, and a variety of morphological changes, including cardiac hypertrophy, myofibril depletion, interstitial fibrosis, and microangiopathy [9–12].

Histone acetyltransferases (HAT) and HDAC have recently garnered attention because they have emerged as important mechanisms in the regulation of a variety of cellular responses. Histone acetylation is mediated by HAT, which results in the modification of the structure of chromatin leading to nucleosomal relaxation, and altered transcriptional activation. In contrast, the reverse reaction is mediated by HDACs which induce deacetylation, chromatin condensation, and transcriptional repression. The acetylation status of histone tails is determined by the interplay between HATs and HDACs [13–15]. Recent observation indicates that regulation of acetylation status mediates proteolytic function in diseased myocardium from human and animal models [16]. These recent studies have indicated the critical role of HDACs in modulating myocardial ischemia/reperfusion injury, cardiac hypertrophy, and skeletal myogenesis [17–22]. Inhibition of HDACs using small molecules is regarded to be one of the most promising approaches for many pathological disorders.

Our studies have demonstrated that inhibition of HDACs plays a critical role in the prevention of myocardial damages, mediation of cardiogenesis, and stimulation of myocardial repair [23–27]. In addition, the function of HDAC was reported to be associated with the pathological process in diabetic status and/or heart failure [28, 29]. However, it remains unknown whether HDAC inhibition could produce a protective effect against myocardial dysfunction in diabetic mice. In this present study, we utilized the STZ-induced diabetic model to define whether HDAC inhibition could preserve cardiac performance and attenuate cardiac remodeling.

## Methods

### Animals

Two-month old ICR male mice were purchased from Charles River Laboratories (Wilmington, MA, USA). All animal experiments were conducted under a protocol approved by the Institutional Animal Care and Use Committee of Institute, which conforms to the Guide for the Care and Use of Laboratory Animals published by the US National Institutes of Health (NIH Publication No. 85-23, revised 1996).

### Experiment protocols

Two month-old mice were made diabetic by intraperitoneal injection of a single dose of freshly prepared STZ solution (200 mg/kg body wt dissolved in citrate buffer, pH 4.5) following overnight fasting [30]. Diabetic status was determined by measuring blood glucose concentration. Another group of mice was injected with vehicle (0.1 mol/l citrate buffer, pH 4.5) to serve as a control. Mice were randomly divided into four groups: *Control* group: mice only received an injection of vehicle (citrate buffer); *Control* *+* *NaBu* group: mice received sodium butyrate (1%), a specific HDAC inhibitor, in drinking water on a daily basis; *STZ-*treated group: mice received intraperitoneal injection of STZ; *STZ* *+* *NaBu* group: mice received intraperitoneal injection of STZ injection followed by sodium butyrate (1%), in drinking water on a daily basis. Tail vein blood glucose samples were measured using One Touch II Glucometer (Lifescan, Inc., Milpitas, CA, USA) to confirm the induction of diabetes. All animals were euthanized at 21 weeks after injection of STZ.

### Echocardiography

Echocardiographic parameters were accessed before and 7, 14, and 21 weeks after the sodium butyrate treatments. Echocardiography was performed to evaluate left ventricular (LV) functions using an Acuson Sequoia C512 system (Siemens Helathcare, PA, USA) equipped with a 15L8 linear array transducer. Mice were placed in supine position on a heating pad after being anesthetized with 1.5% isoflurane mixed with oxygen. Pre-warmed ultrasound gel was applied on the chest throughout the measurements. At the signal depth of 25 mm, 2-D B-mode and M-mode images were recorded on short axis views at the level of the papillary muscles. The following parameters were measured on the M-mode tracings and averaged from 3 to 6 cardiac cycles: left ventricular internal dimension-diastole (LVID;d), left ventricular internal dimension-systole (LVID;s), ejection fraction (EF), fraction shortening (FS), left ventricular posterior wall thickness in end-diastole (LVPW;d), and end-systole (LVPW;s). Data were calculated with accompanying software.

### Immunohistochemistry

Tissue sections were de-paraffinized for 30 min at 70°C and subsequently immersed in xylene and ethanol at decreasing concentration. Immunostaining was performed as described previously [27]. All de-paraffinized tissue sections went through antigen retrieval by boiling of slides at 100°C for 1 h. Wheat germ agglutinin (WGA) staining was carried out to measure cell size. The outline of myocytes was traced in the LV of each animal, using NIH Image J software to determine myocyte cross-sectional area. A value from each heart was calculated by the measurements of approximately 400–600 cells in a remote area from 5 randomly selected image areas in an individual heart. For evaluation of interstitial fibrosis, Picrosirius red staining of cardiac sections was conducted. Interstitial collagen was examined from five randomly selected regions from each tissue section using an Olympus BX51 microscope (Olympus, Center Valley, PA, USA). For α-smooth muscle actin (α-SMA) and cluster of differentiated CD31 immunostaining, cardiac sections were incubated with primary antibodies including CD31 (Millipore, Billerica, MA, USA) and anti-α-SMA (Sigma, St. Louis, MO, USA) overnight at 4°C. Signals were visualized by incubation with the corresponding secondary antibodies including goat-anti-rat-Cy3 and goat-anti-mouse-Cy3 (Life Technologies, Carlsbad, CA, USA) at room temperature for 1 h. Nuclei were stained with 4′, 6-diamidino-2-phenylindole (DAPI).

### Terminal deoxynucleotidyl transferase dUTP nick end labeling (TUNEL) assay

De-paraffinized sections were processed for a TUNEL assay using a TACS^®^ TdT In Situ Apoptosis Detection Kits (Trevigen, Gaithersburg, MD, USA) following the manufacturer’s instructions. TUNEL positive cells were observed using confocal laser scanning microscopy LSM 700 (Carl Zeiss). The numbers of TUNEL positive cells were determined and were normalized to the tissue area. DAPI was used to counter stain for nuclei.

### Immunoblotting and immunoprecipitation

Heart tissues were homogenized on ice in RIPA buffer with Halt protease and phosphatase inhibitor cocktail. Proteins (50 µg/lane) were resolved by SDS gel and transferred onto PVDF membranes. The membranes were then blocked with 5% powdered milk in TBST for 1 h and were subsequently probed with primary antibodies overnight at 4°C. The following primary antibodies were used in this study: HDAC4, acetylated-Lysine, phosphorylated p38, and active casepase-3 from Cell Signaling (Danvers, MA, USA); GLUT 1, GLUT4, p38, SOD1, and β-actin were from Santa Cruz Biotechnology (Dallas, TX, USA). Horseradish peroxidase-conjugated monoclonal antibodies (1: 2,000) were used for chemiluminescence detection.

For immunoprecipitation, samples were pre-cleared prior to immunoprecipitation to reduce the amount of non-specific contaminants. The EZView red protein A affinity gel (Sigma-Aldrich, St.Louis, MO, USA) was incubated with myocardial lysates for 60 min at 4°C. Samples were centrifuged, and supernatants were obtained. Proteins were incubated with the indicated primary antibodies at 4°C overnight. Beads were added to lysate plus antibody mix, and proteins were further incubated for 2 h at 4°C. In addition, IgG was also used as immunoprecipitation control and non-immunoprecipitated lysate was used as a control for detected molecular weight. After incubation, samples were washed with RIPA buffer five times, and proteins were eluted with 4× loading buffer by boiling and subjected to SDS-PAGE.

### HDAC activity measurement

Myocardial HDAC activity was measured as described preciously in detail [25].

### Statistical analysis

All data are expressed as mean ± SEM. Differences among multigroups were analyzed by one-way analysis of variance (ANOVA), followed by Bonferroni correction. A probability of *p* < 0.05 was considered to be a significant difference.

## Results

### Induction of hyperglycemia

**Fig. 1:**
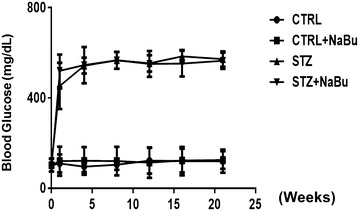
The time course of measuring the concentration of blood glucose. The peripheral blood concentration was measured on weekly basis. After overnight fasting, blood samples were collected from the tail tip vein on mice. Measurements were performed on control, STZ and STZ+ Sodium butyrate treatment groups, respectively. Mice with blood glucose readings of >250 mg/dl were diagnosed diabetic. Values represent mean ± SEM (n = 5–7 per group); *CNTL* control; *STZ* streptozotocin; *NaB* sodium butyrate.

A single injection of STZ (200 mg/kg) was used to induce diabetes in ICR mice. At 1 week after the injection, the fasting blood glucose levels of >450 mg/dl were detected in all mice that received STZ injection. Approximately 20% mice did not survive to sustain the toxicity of STZ by day 14. The surviving mice had blood glucose levels of >450 mg/dl throughout the study period. As shown in Fig. 1, sodium butyrate treatment did not lower the blood glucose level. The two diabetic groups showed no difference in blood glucose level by the end of the study period.

### Ventricular function

**Fig. 2:**
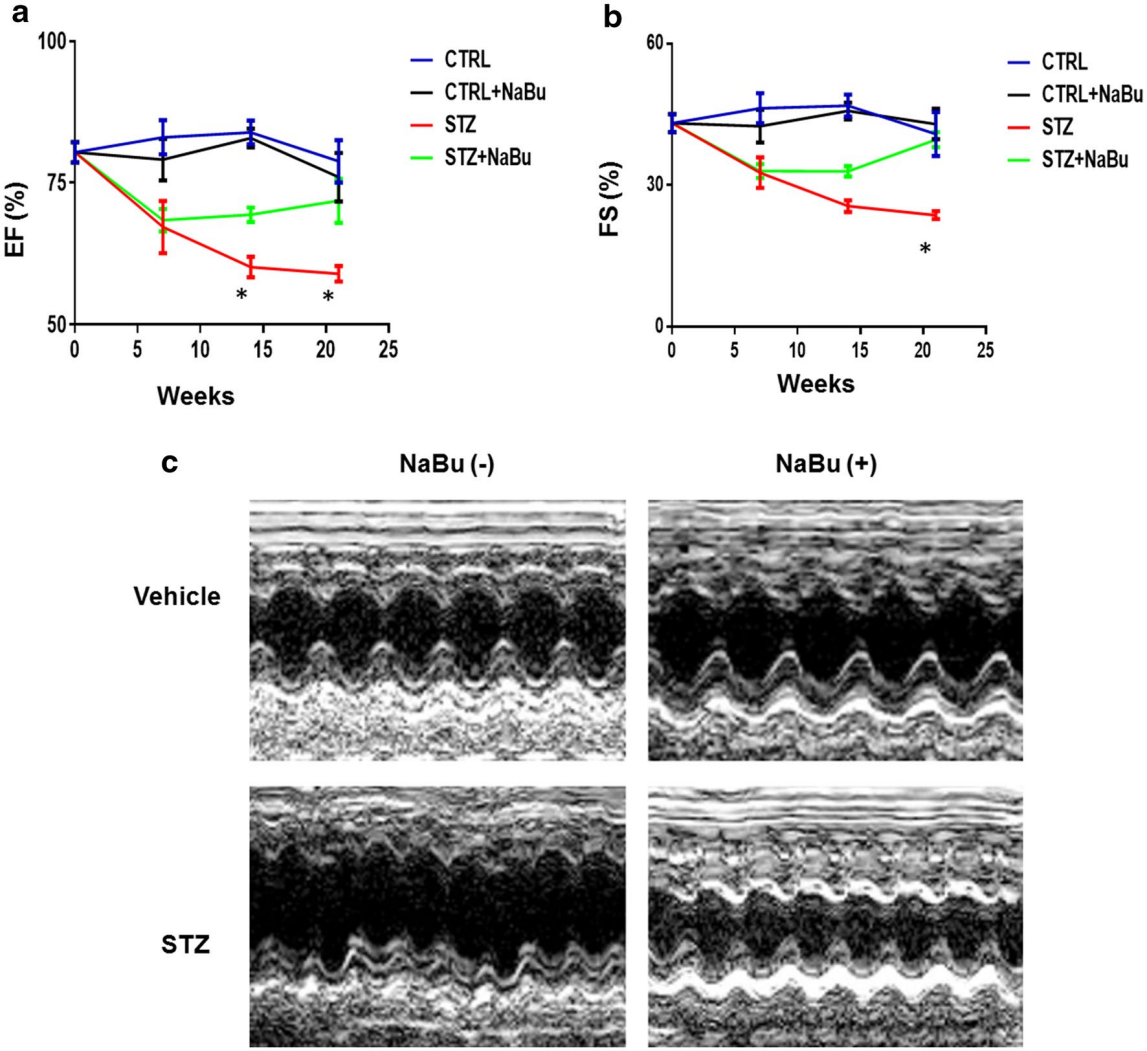
Effects of HDAC inhibition on cardiac function in STZ-induced diabetic heart. Echocardiographic measurements of ventricular functional parameters includes: **a** Ejection fraction; **b** fractional shortening; **c** Representative echocardiographic M-mode images. Values are shown as mean ± SEM (n = 5–7 per group); *p < 0.05 vs STZ+ NaBu. *NaBu* sodium butyrate.

Serial echocardiography was performed immediately before and up to 21 weeks after treatments. As shown in Fig. 2, STZ induced diabetic mice displayed cardiac dysfunction, as indicated by the reduction of both EF and FS as compared to Control group. However, administration of sodium butyrate resulted in improvements in EF and FS as compared with STZ group (Fig. 2a, b). The representative images of M-mode among groups are shown in Fig. 2c. Furthermore, an increase in left ventricular internal dimension (LVID) was observed in STZ-treated group as compared with Control and Control + NaBu groups starting at 7 weeks following STZ injection throughout the 21-week period. LVID;s was significantly reduced in sodium butyrate-treated STZ mice as compared with STZ diabetic mice without treatment (Additional file 1: Table S1). In addition, STZ-induced diabetic mice demonstrated an increase in LVPW, which was also attenuated by treatment of animals with sodium butyrate (Additional file 1: Table S1).

### Cardiac remolding and interstitial collagen deposition

**Fig. 3:**
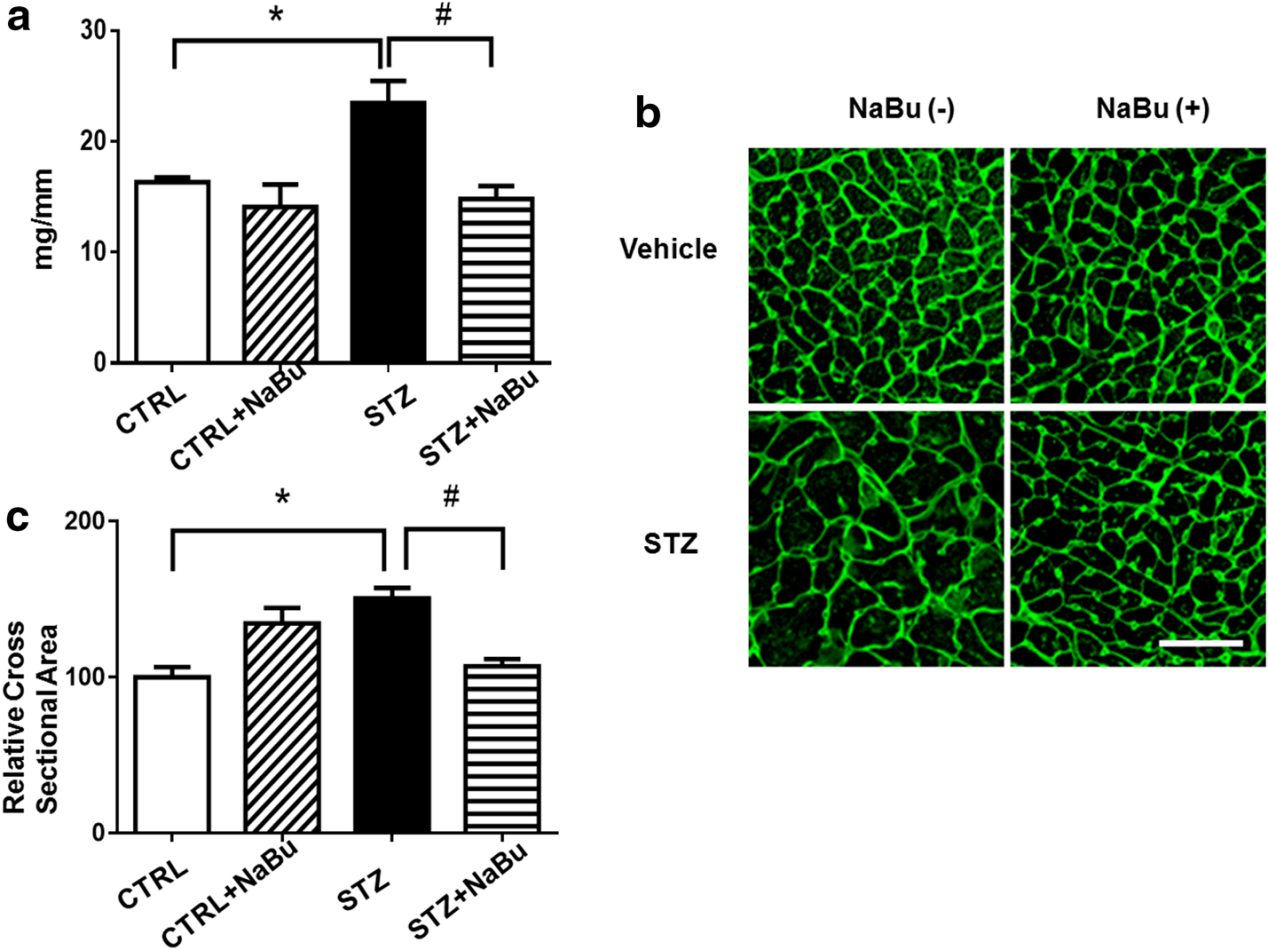
Effects of HDAC inhibition on attenuating cardiac remodeling in STZ-induced diabetic heart. **a** The ratio of heart weight to tibia length. **b** Representative images of WGA staining in the MI hearts. **c** Quantitative analysis of myocyte cross-sectional area. Values are shown as mean ± SEM (n = 5 per group); *p < 0.05 vs CTRL, ^#^ *p* < 0.05 vs STZ+ NaBu. *NaBu* sodium butyrate. *Scale bar* 100 µm.

**Fig. 4:**
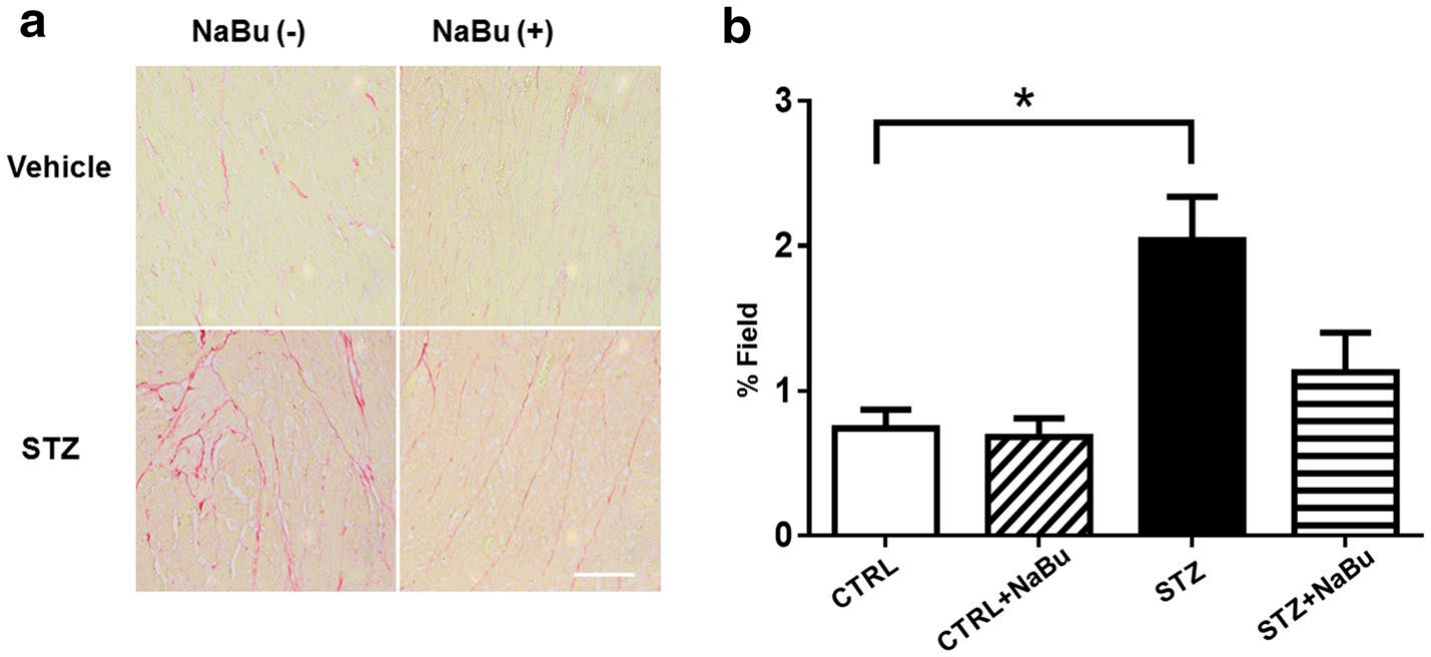
Effect of HDAC inhibition attenuates interstitial collagen deposition in STZ-induced diabetic heart. **a** Representative images of histological picro-sirius red staining. **b** Quantitative analysis of myocyte interstitial collagen deposition. Values are shown as mean ± SEM (n = 5 per group); *p < 0.05 vs CTRL. *NaBu* sodium butyrate. *Scale bar* 100 µm

The ratio of heart weight to tibia length was used to evaluate the hypertrophic response. STZ injection resulted in an increase in the heart weight/tibia length ratio as compared to control hearts. Treatment of STZ mice with sodium butyrate significantly reduced the heart weight/tibia length ratio (Fig. 3a). In addition, STZ treatment caused a significant increase in heart weight/body weight ratios (Additional file 1: Table S2), which was prevented by treatment with sodium butyrate. *STZ-induced diabetic mice* WGA staining was performed to access the cross sectional cardiomyocyte size. As shown in Figs. 3b, c, the STZ-treated mice showed an increase in cross-sectional cardiomyocyte diameters as compared with control groups. However, the cross-sectional cardiomyocyte diameter was significantly reduced in STZ mice receiving sodium butyrate as compared with STZ mice only (Fig. 3c). As shown in Fig. 4a, b, the collagen content was significantly increased in STZ group as compared with control groups, but administration of sodium butyrate led to a reduction in interstitial collagen deposition in the myocardium as compared with STZ-treated mice alone although there is no statistically difference between the two diabetic groups.

### Inhibition of HDACs resulted in up-regulation of GLUT1 and 4 and acetylation of GLUT1

**Fig. 5:**
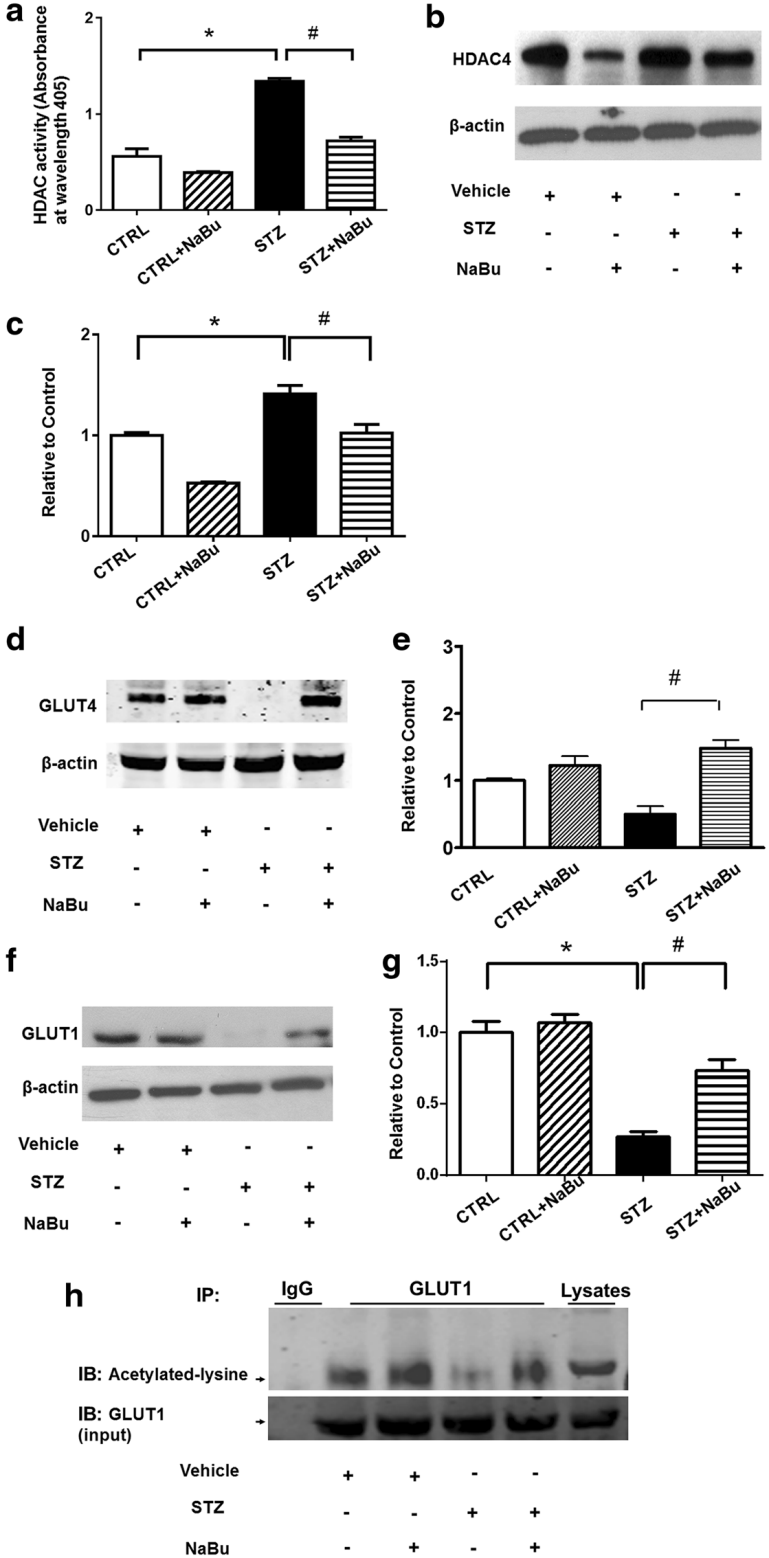
HDAC inhibition decreases HDAC activity and increases GLUT1 and GLUT4. **a** Measurement of HDAC activity in diabetic myocardium from different groups; **b** representative Western blot of HDAC4 proteins in myocardium; **c** densitometric analysis of Western blot of HDAC4 protein levels; **d** representative Western blot of GLUT4 proteins in myocardiums; **e** densitometric analysis of GLUT4 proteins in myocardium; **f** representative Western blot of GLUT1 proteins in myocardium; **g** densitometric analysis of GLUT1 proteins in myocardium; **h** GLUT1 acetylation in diabetic myocardium. *IB* immunoblot; *IP* immunoprecipitation; *IgG* serves as IP control; lysates from myocardium serve as signal control of detected proteins. The densitometric signal was normalized to the control group and expressed as a percentage. Values are shown as mean ± SEM (n = 3 per group); *p < 0.05 vs CTRL, ^#^ *p* < 0.05 vs STZ+ NaBu. *NaBu* sodium butyrate.

**Fig. 6:**
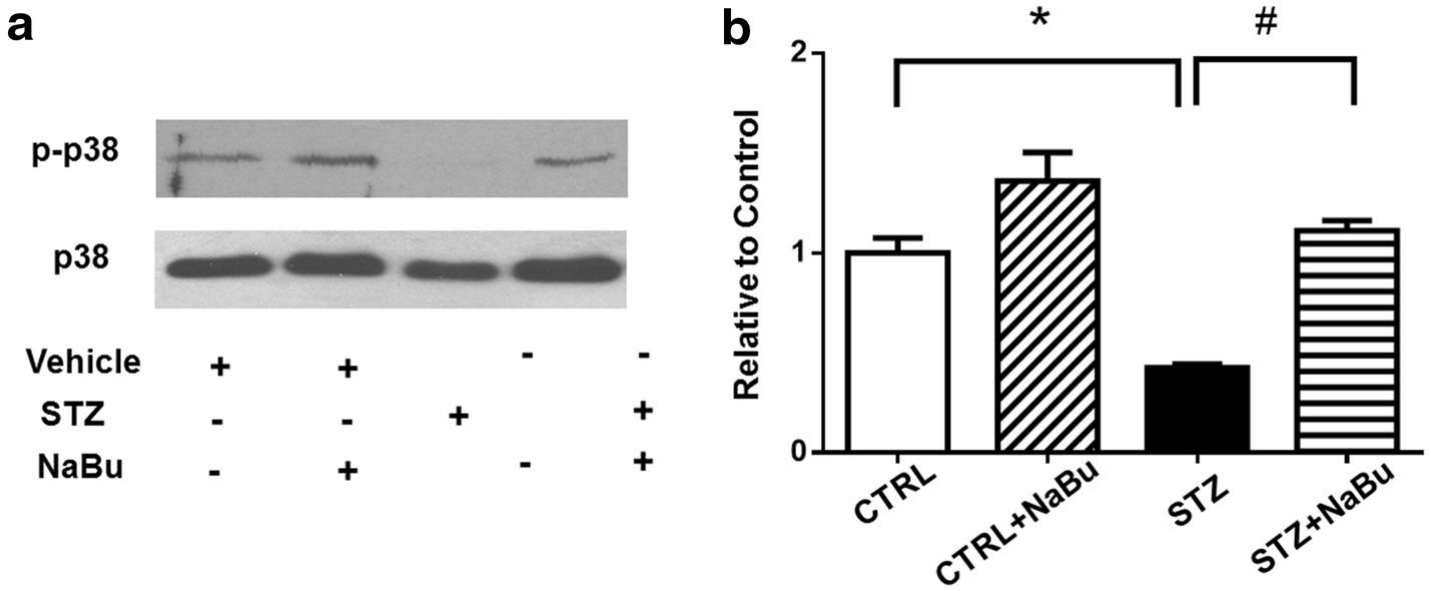
HDAC inhibition increased p38 phosphorylation in diabetic myocardium. **a** Representative Western blot showing phosphorylated p38 and p38 proteins. **b** Densitometric analysis of phosphorylated p38 protein level in different groups. The densitometric signal was normalized to the control group and expressed as a percentage. Values are shown as mean ± SEM (n = 3 per group); *p < 0.05 vs CTRL, ^#^ *p* < 0.05 vs STZ+ NaBu. *NaBu* sodium butyrate.

When we measured HDAC activity in the diabetic myocardium, HDAC activity demonstrated an increase in STZ-induced diabetic heart, but HDAC activity was significantly reduced following the treatment of animals with sodium butyrate (Fig. 5a). In addition, as shown in Fig. 5b, c, treatment of mice with sodium butyrate decreased the expression level of HDAC4 in both Control and STZ-treated groups. Other HDAC isoforms were not changed in the diabetic myocardium between groups (Additional file 1: Figure. S1). The glucose transporter signaling pathway was further confirmed. As shown in Fig. 5d–g, STZ induced a down-regulation of GLUT1 and GLUT4 protein levels in STZ heart as compared with Control groups. However, HDAC inhibition resulted in up-regulations in both GLUT4 and GLUT1 in STZ heart, which restored the signal to the same level as the control group. Notably, GLUT1 was subjected to regulation by acetylation. As shown in Fig. 5h, HDAC inhibition increased the level of acetylated GLUT1 in sodium butyrate-treated STZ mice as compared with STZ alone. However, HDAC inhibition did not result in a significant increase in acetylated GLUT4 although we noted there was a detectable level of acetylated GLUT4. Interestingly, as shown in Fig. 6, we found that the diabetic myocardium demonstrated a decrease in phosphorylated p38 level, but HDAC inhibition resulted in a significant increase in phosphorylation of p38.

### Anti-apoptotic effects of HDAC inhibition in the diabetic myocardium

**Fig. 7:**
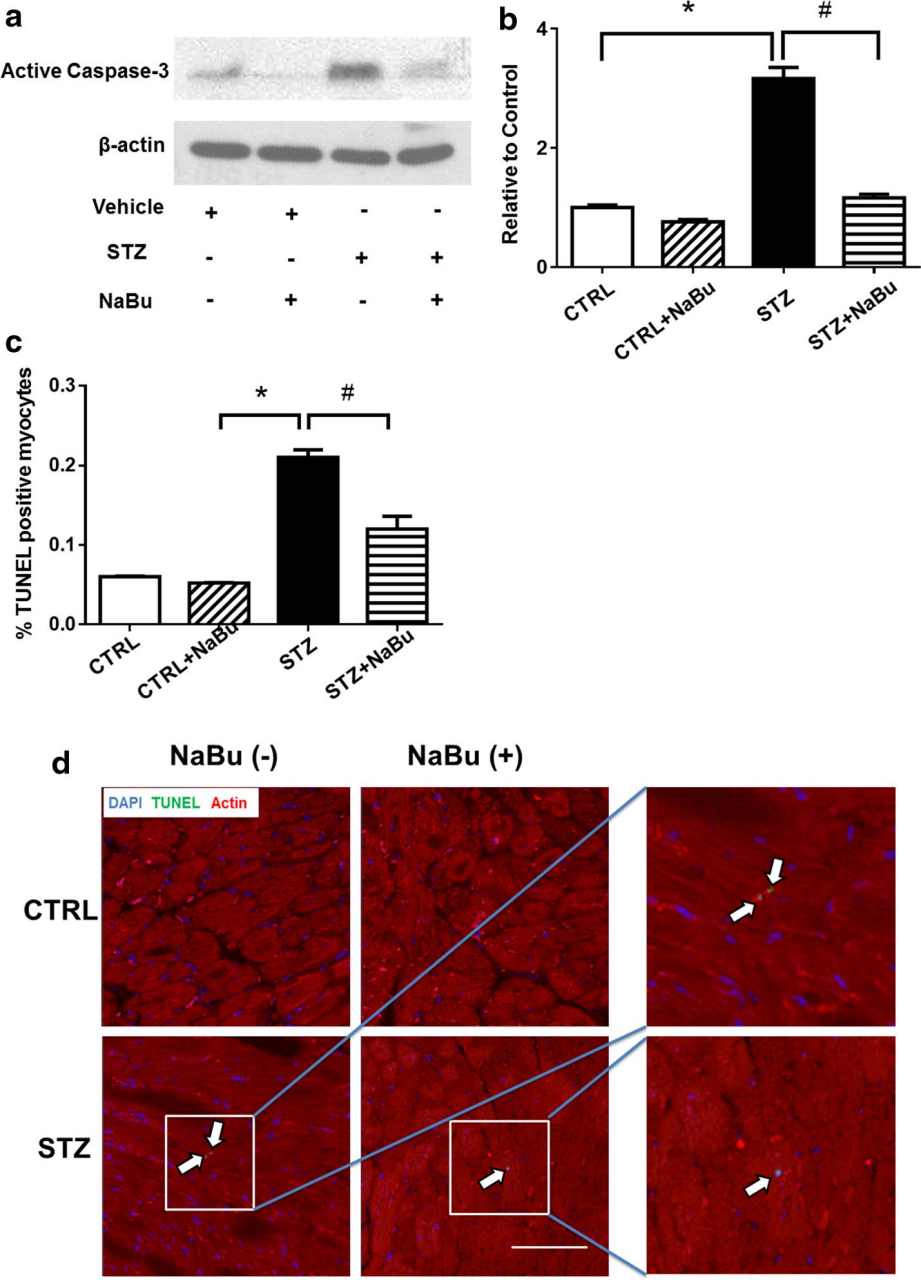
HDAC inhibition reduced apoptosis in STZ-induced diabetic heart. **a** Representative Western blots of active caspase-3. **b** densitometric analysis of active caspase-3 proteins. **c** Quantitative analysis of terminal deoxynucleotidyl transferase (TdT)-mediated dUTP nick end labeling (TUNEL)-positive nuclei in myocardium in different groups. **d** Representative images of TUNEL stainings in myocardial sections. Nuclei were stained in *blue* (DAPI) and cardiomyocytes in *red* (α-sarcomeric actinin); TUN EL-positive nuclei were stained in *green.* *α*-*Sarc Act* α-sarcomeric actinin; Values are shown as mean ± SEM (n = 3 per group for Western blots, n = 5 per group for TUNEL); *p < 0.05 vs CTRL, ^#^ *p* < 0.05 vs STZ+ NaBu. *NaBu* sodium butyrate. *Scale bar* 100 µm.

The apoptotic marker active caspase-3 was examined in the myocardium. As shown in Fig. 7a, active caspase-3 was increased in STZ group as compared with samples from Control groups. Densitometric analysis confirms that the administration of sodium butyrate significantly reduced activated caspase-3 in STZ mice (Fig. 7b). As shown in Fig. 7c, d, TUNEL analysis indicates that STZ-injection increased the number of TUNEL-positive nuclei, but HDAC inhibition reduced apoptotic signals in the diabetic heart.

### HDAC inhibition increases endogenous antioxidant enzyme SOD1

**Fig. 8:**
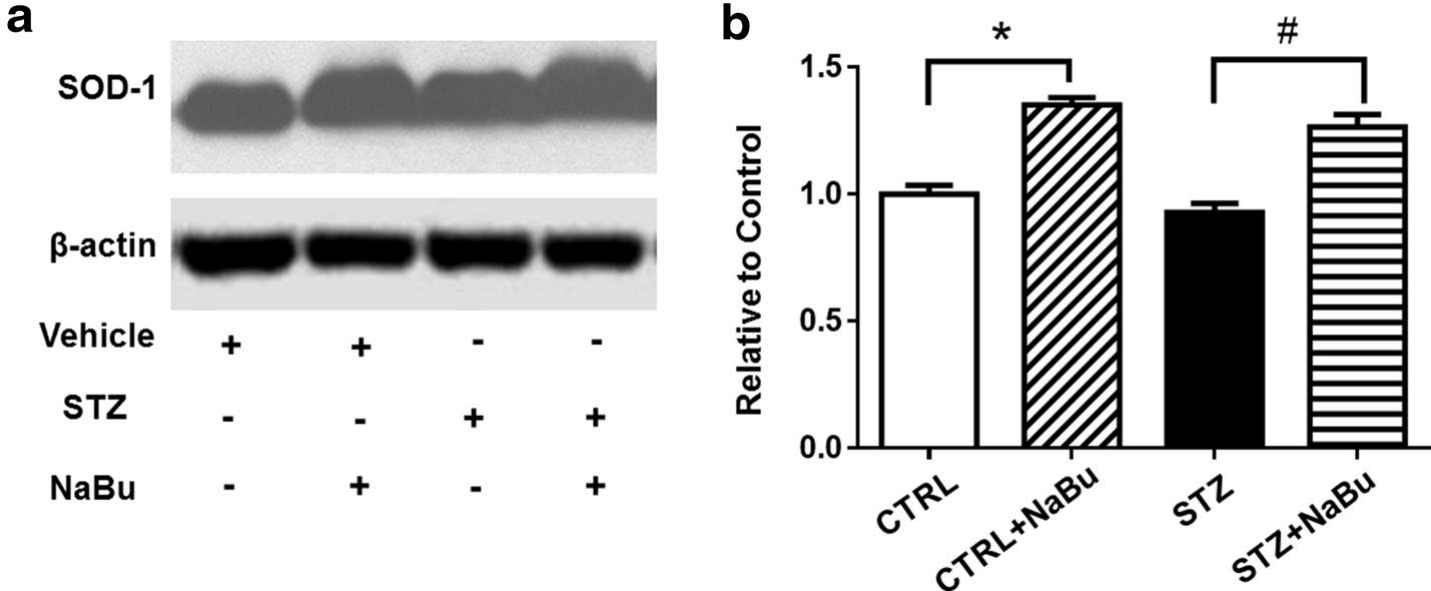
HDAC inhibition reduced SOD1 expression in myocardium. **a** Representative Western blots of active SOD1. **b** Densitometric analysis of Western blot results. Values are shown as mean ± SEM (n = 3 per group); *p < 0.05 vs CTRL, ^#^ *p* < 0.05 vs STZ+ NaBu. *NaBu* sodium butyrate.

Superoxide dismutase has been implicated in protective effects against myocardial ischemic injury. The levels of SOD1 in the myocardium were examined. As shown in Fig. 8a, all mice treated with sodium butyrate showed an increase in SOD1 level as compared to mice without sodium butyrate treatment. Densitometric analysis also indicates significant increases in SOD1 1evel following HDAC inhibition (Fig. 8b). In addition, the diabetic myocardium demonstrated an increase in superoxide production, which was suppressed by HDAC inhibition (Additional file 1: Figure S2).

### HDAC inhibition induces angiogenesis in the diabetic myocardium

**Fig. 9:**
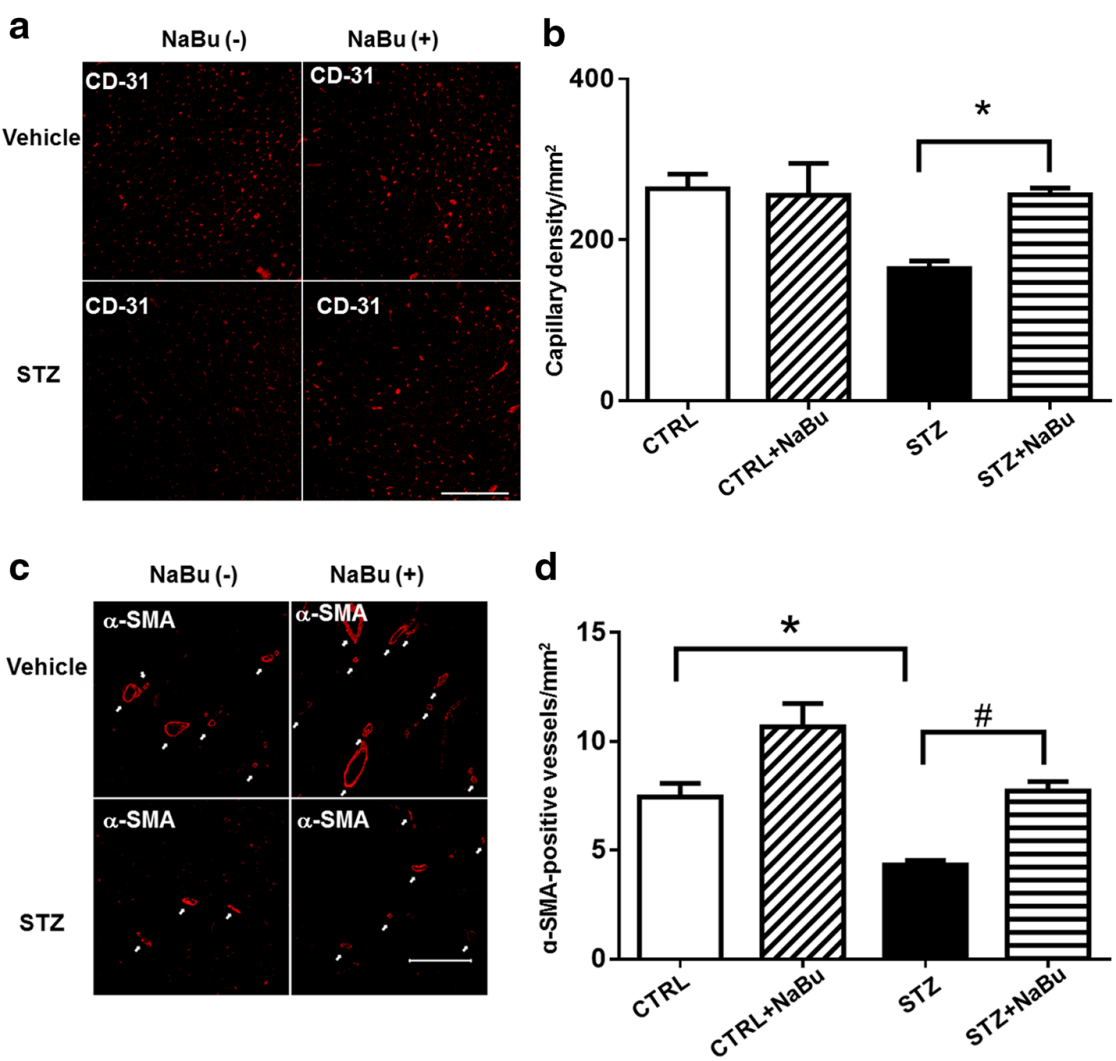
HDAC inhibition promotes angiogenesis in STZ-induced diabetic heart. **a**, **c** Representative images of CD31 and α-SMA staining, respectively. **b**, **d** quantitative analysis of angiogenetic response. Results were indicated by the *number* of CD31- or α-SMA-positive cells per mm^2^. Values are shown as mean ± SEM (n = 3 per group); *p < 0.05 vs CTRL, ^#^ *p* < 0.05 vs STZ+ NaBu; *NaBu* sodium butyrate. *Scale bar* 100 µm for CD31, 200 µm for α-SMA.

As shown in Fig. 9a, CD31 positive capillary density was decreased in STZ mice. Administration of sodium butyrate substantially increased the capillary density in myocardium from STZ mice. Furthermore, we examined α-SMA positive microvessels in STZ-induced diabetic mouse hearts. Figure 9c shows α-SMA staining in the heart sections from all treatment groups. There is a significant decrease in α-SMA-positive microvessels in STZ mice as compared to Control group, but HDAC inhibition resulted in a marked increase in microvessels. As shown in Fig. 9b, d, HDAC inhibition increased CD31 and α-SMA positive vessels in the diabetic myocardium.

## Discussion

### Salient findings

Our study is the first to document that HDAC inhibition preserves cardiac performance and suppresses cardiac remodeling in diabetic cardiomyopathy. Specifically, (1) Serial echocardiographic evaluation indicates that HDAC inhibition resulted in the preservation of ventricular function in STZ-induced diabetic heart; (2) HDAC inhibition plays a profound effect in suppressing interstitial fibrosis and attenuating myocyte hypertrophy in the diabetic myocardium; (3) HDAC inhibition mitigated the frequencies of apoptosis in the diabetic myocardium by decreasing active caspase 3 and TUNEL positive signals; (4) HDAC inhibition resulted in the increase of SOD1, GLUT1, and GLUT4 protein levels in diabetic hearts, and acetylation of GLUT1 was elevated following HDAC inhibition; (5) Diabetic hearts exhibited significant decreases in CD31 and α-SMA positive microvessels, which was prevented following HDAC inhibition.

Recent evidence has indicated a genetic association between diabetes and HDACs. HDAC inhibitors promote β-cell development and function that positively affect diabetic microvascular complications [31]. Ventricular hypertrophy in diabetic hearts was mitigated by inhibition of HDACs [32]. Transient hyperglycemia is considered to promote gene-activating epigenetic changes critical in the progression of vascular complications [33]. Therefore, the function of epigenetics in the development of diabetes is largely recognized [34]. However, its implications in diabetes-associated cardiomyopathy remain to be determined. Our study showed that HDAC inhibition attenuates progressive dysfunction, suggesting that HDACs play an important role in controlling the progression of heart failure.

Clinical studies demonstrate that diabetes mellitus increased the susceptibility of the myocardium to ischemic injury [35, 36]. In the present study, when we employed a STZ-induced diabetic model [37], we have demonstrated that the diabetic myocardium (DM) presents with progressive LV systolic failure following STZ treatment for 21 weeks, which is consistent with observations in the development of cardiac dysfunction in STZ-induced diabetic rats [38]. This was supported by an observation that HDAC inhibition prevented the heart from effects of diet-induced obesity and insulin resistance in mouse models [39]. Further estimation of diastolic function in this model will determine whether diabetic cardiomyopathy developed. In addition, the low dose of STZ could be used as a diabetogenic method to induce diabetes to avoid the high rate of mortality caused by high dose of drug. Treatment of sodium butyrate also decreased HDAC activity and HDAC4 levels in DM myocardium, which is in agreement with our recent observations that specific inhibition of HDAC4, by reducing HDAC4 protein, promotes stem cell-derived myocardial repair [40]. Furthermore, we could not find significant changes in other HDAC isoforms following HDAC inhibition in diabetic myocardium. Myocardial enhancing factor 2 (MEF2) is reported to be associated with class II HDACs to mediate cardiac growth and remodeling [41]. We found that HDAC inhibition only slightly increased MEF2 protein levels in STZ-induced myocardium (not shown), implying that MEF2 may not be a major target following HDAC inhibition in STZ-induced diabetic myocardium. An important pathological feature of diabetic cardiomyopathy is cardiac hypertrophy [42]. In the initial stage, this hypertrophic cardiomyopathy may be an adaptive response responsible for enhancing cardiac performance [43]. However, sustained hypertrophic growth of the myocardium may be associated with the occurrence of myocardial remodeling [20]. HDAC inhibitors blocked cardiac hypertrophy induced by angiotensin II infusion and aortic banding (18.19). We observed that HDAC inhibition remarkably prevented these hypertrophic features in DM. The anti-hypertrophic effect of HDAC inhibition in the DM heart is well mirrored by our recent studies in which cardiac hypertrophy was mitigated in the infarcted hearts following global HDAC inhibition or infarcted hearts engrafted with cardiac stem cells treated with HDAC inhibitors [27].

Myocardial fibrosis is another important hallmark of diabetic cardiomyopathy, and it is also featured by the accumulation of interstitial collagens in the hearts [44]. The present results demonstrate that interstitial collagen in DM hearts was reduced after treatment with HDAC inhibition, indicating the function of HDAC in preventing interstitial fibrosis. Another finding is an increase of myocyte apoptosis in DM hearts. Although the significance of myocyte apoptosis in diabetic myocardium remains speculative, progressive loss of myocytes could exacerbate cardiac dysfunction and structure deterioration.

We reported that HDAC inhibition protects the heart against ischemia/reperfusion through blocking of reactive oxidant species [25]. Oxidative stress has been suggested by several studies to underlie hyperglycemia-induced myocardial cell deaths [45–47]. High glucose-induced cardiomyocyte apoptosis is associated with the generation of reactive oxygen species [48], and normalization of SOD1 activity was associated with consequent bolstering of anti-oxidant defenses in diabetes [49].The present study shows that SOD1 was markedly increased by HDAC inhibition, suggesting that the increased anti-oxidant stress elicited by HDAC inhibition accounts for the suppression of myocardial remodeling in the diabetic heart. In addition, the production of superoxides was increased in the diabetic myocardium, but HDAC inhibition attenuated the superoxide production of diabetic hearts, revealing a link between HDAC inhibition and suppression of superoxide.

It is known that pathological cardiac hypertrophy with reduced contractility is accompanied by impaired coronary angiogenesis [50]. The reductions in capillary and arteriolar densities were shown in the myocardial infarction model of diabetic conditions [51, 52]. Our study demonstrates a significant reduction in microvessel density in diabetic myocardium, but vascular growth was stimulated by treatment with HDAC inhibition. This increase in microvessel density following HDAC inhibition in diabetic myocardium may also be attributable to the improvement in cardiac function and attenuation of remodeling.

Two glucose transporter proteins (GLUT1 and GLUT4) were reported to be reduced in the diabetic myocardium [53]. We have shown that GLP-1-induced myocardial protection is associated with the increase of GLUT4 [53]. In this study, both GLUT1 and GLUT4 were decreased in the diabetic myocardium, but the decrease of both GLUT1 and GLUT4 was prevented by HDAC inhibition. Interestingly, our results show that acetylation of GLUT1 was increased by HDAC inhibition. However, HDAC inhibition did not result in a significant change in the acetylation of GLUT4 (data not shown). This suggests that acetylation of GLUT1 and GLUT4 may respond differently following HDAC inhibitions or depend on the magnitudes of HDAC inhibition. Although the content of GLUT1 and GLUT4 were increased by HDAC inhibition in the diabetic animals, blood glucose level was not reduced by HDAC inhibition in this studies. It is likely that HDAC inhibition could not reduce the peripheral glucose concentration, but instead modulate insulin resistance in the diabetic status, which will be an interesting subject to investigate in the future. It is interesting to elucidate whether acetylation of GLUT1 could mediate its physiological functions in the future. In addition, we have shown that p38 phosphorylation was associated with cardioprotection induced by glucagon-like peptide (GLP-1) in myocardial ischemia and reperfusion [54]. The present study again reveals that HDAC inhibition increased p38 phosphorylation in diabetic hearts. The future study may attempt to document whether there exists a direct relationship between p38 phosphorylation and GLUT1 acetylation in diabetic hearts. It is not clear whether the PI3 kinase/Akt-1 signaling pathway is also involved in the protective effects induced by HDAC inhibition. On the other hand, mitochondrial oxygen consumption was impaired in the diabetes related mouse model [55].Our previous observation also indicates that stimulation of GLP-1R could increase mitochondrial oxygen consumption in myoblasts exposed to hypoxia/reoxygenation [56]. It is not clear whether HDAC inhibition could also modulate mitochondrial oxygen consumptions in this observation. It has been reported that pathological stress activates the chromatin repressor complex containing HDAC to inhibit the transcription of Mhrt, a long noncoding RNA in the heart [57]. It would be interesting to define whether long non-coding transcripts would also involve the protective effects of HDAC inhibitors in the prevention of diabetic pathology. In this study, we utilized a high dose of STZ to induce the diabetic model. Because the dose of STZ that was used could be cytotoxic in the animal strain, this could be associated with the high mortality rate and abundant myocyte death during the development of diabetes. This could complicate the type 1 diabetic phenotype. This is a limitation for this observation.

## Conclusion

Our study is the first to document that HDAC inhibition attenuated diabetic cardiomyopathy by improving ventricular function, attenuating remodeling, and suppressing cardiac hypertrophy. The protective effects of HDAC inhibition in the diabetic myocardium are closely associated with decreased apoptosis, stimulation of endogenous angiogenesis, an increase of anti-oxidant SOD1, and activations of GLUT1 acetylation and p38 phosphorylation. HDAC inhibition provides a novel approach to the treatment of patients with diabetic cardiomyopathy. Our study indicated that inhibition of HDACs effectively prevented cardiac dysfunction and cardiac remodeling in the diabetic heart, which is related to the promotion of angiogenesis, anti-apoptosis, and acetylation of GLUT1.

### Perspectives and translational implication

Diabetes mellitus threatens to become a global health crisis; treatment of diabetes and its implications constitutes a major health care expenditure. A significant proportion of diabetic patients are known to develop diabetic cardiomyopathy, which is one of the leading causes of increased morbidity and mortality in patients with diabetes mellitus. HDAC regulates transcriptional changes and plays a critical role in mediating myocardial development and disease. From both a mechanistic and a translational point of view, the pathway identified in the present study will provide a strong foundation on which HDAC inhibition may serve as the novel therapeutic target to prevent the progression of diabetes-induced cardiac dysfunction. HDAC inhibitors are well tolerated in humans, and clinical trials investigating their efficacy in anti-cancer therapy are currently underway. Therefore, the therapeutic merit of HDAC inhibition in treating diabetic cardiomyopathy is potentially important. Exploration of the functional role of HDAC inhibition and evaluation of its clinical outcome will provide direct evidence to support a potential clinical implication.

## Additional file

Additional file 1:
**Table S1.** Echocardiographic parameters among the different groups. **Table S2.** Heart weight, body weight and heart/body ratio. **Figure S1.** Other HDAC isoforms in myocardium from STZ-treated mice and treatments. **Figure S2.** HDAC inhibition decreased the production of superoxide in myocardium from STZ-induced diabetic mice and different treatments.

## Abbreviations

HDACs: histone deacetylases
HATs: histone acetyltransferases
DM: diabetic myocardium
STZ: streptozotocin
NaBu: sodium butyrate
LV: left ventricular
GLUT: glucose transporter type
LVID;d: left ventricular internal dimension-diastole
LVID;s: left ventricular internal dimension-systole
EF: ejection fraction
FS: fraction shortening
