# Supplementary material for: Histone deacetylase (HDAC) inhibition improves myocardial function and prevents cardiac remodeling in diabetic mice
Source: Cardiovasc Diabetol. 2015 Aug 7;14:99. doi: 10.1186/s12933-015-0262-8 (PMC4527099; doi:10.1186/s12933-015-0262-8)
Supplement: Additional file 1: — Table S1. Echocardiographic parameters among the different groups. Table S2. Heart weight, body weight and heart/body ratio. Figure S1. Other HDAC isoforms in myocardium from STZ-treated mice and treatments. Figure S2. HDAC inhibition decreased the production of superoxide in myocardium from STZ-induced diabetic mice and different treatments. [file 12933_2015_262_MOESM1_ESM.docx]

**Supplemental Materials and Methods**.

**Measurement of superoxide production in myocardium**. Superoxide production in myocardial tissues was measured with lucigenin-enhanced chemiluminescence according to the descriptions with the modification (**1**). Briefly, cardiac lysates (20 μg) was suspended in 200 μl of an assay buffer composed of 100 mmol/l potassium phosphate (pH 7.0), 10 μmol/l flavin adenine dinucleotide (FAD), 1 mmol/l NaN_3_, and 1 mmol/L EGTA. After preincubation with 5 μmol/l lucigenin, NADPH was added to a final concentration of 100 μmol/l. Chemiluminescence was continuously monitored using a luminometer. The reaction was terminated by addition of SOD (100 μg/ml). Superoxide productions were determined by the measurement of arbitrary light units and expressed as a percentage of mean arbitrary light units per minute in the control group.

**Reference**:

[See comment in PubMed Commons below](http://www.ncbi.nlm.nih.gov/pubmed/22982779#comments)

1.[Zhao TC](http://www.ncbi.nlm.nih.gov/pubmed/?term=Zhao%20TC%5BAuthor%5D&cauthor=true&cauthor_uid=22982779), [Zhang L](http://www.ncbi.nlm.nih.gov/pubmed/?term=Zhang%20L%5BAuthor%5D&cauthor=true&cauthor_uid=22982779), [Liu JT](http://www.ncbi.nlm.nih.gov/pubmed/?term=Liu%20JT%5BAuthor%5D&cauthor=true&cauthor_uid=22982779), [Guo TL](http://www.ncbi.nlm.nih.gov/pubmed/?term=Guo%20TL%5BAuthor%5D&cauthor=true&cauthor_uid=22982779).Disruption of Nox2 and TNFRp55/p75 eliminates cardioprotection induced by anisomycin. [Am J Physiol Heart Circ Physiol.](http://www.ncbi.nlm.nih.gov/pubmed/22982779) 2012;303(10):H1263-72.

**Supplemental Legends**:

**Supplemental Table 1**. Echocardiographic parameters among the different groups.

**Supplemental Table 2**. Heart weight, body weight and heart/body ratio among the different groups

**Supplemental Figure 1**. Other HDAC isoforms in myocardium from STZ-treated mice and treatments. The Western blot shows that HDAC inhibition did not affect the signals 2, 3, 6 and 10. The details of methods for western blot are described in main text of the manuscript. Primary antibodies including HDACs 2, 3, 6 and 10 were purchased from Cell Signaling ^TM^ (Danvers, MA). The methods of Western blot were described in the main text of the manuscript. The blots represent the two independent experiments.

**Supplemental Figure 2**. HDAC inhibition decreased the production of superoxides in myocardium from STZ-induced diabetic mice and different treatments. Superoxide productions were determined by lucigenin-enhanced chemiluminescence and expressed as a percentage of mean arbitrary light units per minute in the control group. Values are shown as mean ± SEM (n = 4 per group); * *p* < 0.05 vs CTRL, ^#^ *p* < 0.05 vs STZ+NaBu; NaBu.
